# Antidepressive and anxiolytic effects of ostruthin, a TREK-1 channel activator

**DOI:** 10.1371/journal.pone.0201092

**Published:** 2018-08-15

**Authors:** Ancy Joseph, Tran Thi Thu Thuy, Le Tat Thanh, Masayoshi Okada

**Affiliations:** 1 Department of Physiology, Kansai Medical University, Osaka, Japan; 2 Institute of Natural Products Chemistry, Vietnam Academy of Science and Technology, Hanoi, Vietnam; 3 Department of Medical Life Science, College of Life Science, Kurashiki University of Science and the Arts, Kurashiki, Okayama, Japan; University of Modena and Reggio Emilia, ITALY

## Abstract

We screened a library of botanical compounds purified from plants of Vietnam for modulators of the activity of a two-pore domain K^+^ channel, TREK-1, and we identified a hydroxycoumarin-related compound, ostruthin, as an activator of this channel. Ostruthin increased whole-cell TREK-1 channel currents in 293T cells at a low concentration (EC_50_ = 5.3 μM), and also activity of the TREK-2 channel (EC_50_ = 3.7 mM). In contrast, ostruthin inhibited other K^+^ channels, e.g. human ether-à-go-go-related gene (HERG1), inward-rectifier (Kir2.1), voltage-gated (Kv1.4), and two-pore domain (TASK-1) at higher concentrations, without affecting voltage-gated potassium channel (KCNQ1 and 3). We tested the effect of this compound on mouse anxiety- and depression-like behaviors and found anxiolytic activity in the open-field, elevated plus maze, and light/dark box tests. Of note, ostruthin also showed antidepressive effects in the forced swim and tail suspension tests, although previous studies reported that inhibition of TREK-1 channels resulted in an antidepressive effect. The anxiolytic and antidepressive effect was diminished by co-administration of a TREK-1 blocker, amlodipine, indicating the involvement of TREK-1 channels. Administration of ostruthin suppressed the stress-induced increase in anti-c-Fos immunoreactivity in the lateral septum, without affecting immunoreactivity in other mood disorder-related nuclei, e.g. the amygdala, paraventricular nuclei, and dorsal raphe nucleus. Ostruthin may exert its anxiolytic and antidepressive effects through a different mechanism from current drugs.

## Introduction

Anxiety and depression are common mental disorders, for which most patients are treated with medication. However, anxiolytic medicines can lead to tolerance and dependence [1]. In addition, approximately one-third of patients with depression are resistant to current antidepressants, such as serotonin reuptake inhibitors and serotonin-norepinephrine reuptake inhibitors [2]. Therefore, new medicines, for which the mechanism of action is different from current ones, are desired for the treatment of these mental disorders.

Potassium (K^+^) channels play a pivotal role in the regulation of excitability of the central neurons. Among the broad range of K^+^ channel families, the most recently identified family is the two-pore domain K^+^ (K2P) channels responsible for background K^+^ currents, which are also known as leak K^+^ currents [3]. Mammalian K2P channels now include 15 members, one of which is the TWIK-related K^+^ channel, TREK-1. These channels are highly expressed in the central nervous system [4, 5] and are suggested to be involved in mental diseases, i.e. anxiety and depression [6, 7]. For instance, TREK-1-deficient mice showed a depression-resistance phenotype through activation of the dorsal raphe nucleus (DRN), which provides serotonergic innervation [6]. Riluzole, which activates TREK-1 channels in addition to Na^+^ channels and glutamate receptor blockade, showed anxiolytic effects [8]. Therefore, TREK-1 channel activators and blockers are possible candidate for anxiolytic and antidepressive drugs, respectively. TREK-1 channels can be activated or inhibited by several chemical compounds. For example, TREK-1 channels are activated by arachidonic acid, volatile anesthetic (chloroform, diethyl ether, halothane, and isoflurane), and riluzole [9], and inhibited by fluoxetine [10] and bupivacaine [11]. However, TREK-1 modulating activities are only side effects of these compounds, and they have major activities elsewhere, e.g. serotonin uptake inhibition and blockade of Na^+^ channels. Currently, there seems to be no TREK-1-specific compound that can regulate the pharmacological activity of this channel.

Plants cells express K^+^ channels, the structures of which are similar to those of mammalian K^+^ channels [12, 13]. In addition, tropical and semitropical plants also produce compounds that modify K^+^ channel function [14, 15]; therefore, botanical compounds are a promising resource for K^+^ channel modifiers. In this study, we screened a library of botanical compounds, which were isolated from plants in Vietnam, for a modulator of TREK-1 channel activity using whole-cell patch clamp recordings. We identified a TREK-1 activator, ostruthin, which had anxiolytic and antidepressive activities in mice. Ostruthin suppressed stress-induced increases in c-Fos expression in the lateral septum without affecting that in the amygdala or DRN, suggesting a possible difference in the mechanism of action from current drugs.

## Materials and methods

### Purification of ostruthin

The roots of *Paramignya trimera* were collected in Khanh Hoa province Vietnam in 2014 and dried. The material (200 g) was powdered and extracted with methanol at room temperature, and the methanol was evaporated under reduced pressure at 45°C. The crude extract was dissolved in CH_2_Cl_2_ at room temperature with sonication. After solvent evaporation at 40°C, the sample was separated to 7 fractions by silica gel column chromatography, and the fourth fraction was again chromatographed on a silica gel column with an increasing concentration of ethyl acetate mixed with n-hexane (5.0–6.7%). Ostruthin was purified to homogeneity (> 99.1%) according to the chromatogram of A_330 nm_.

### Patch-clamp recordings

For recordings of K^+^ channel currents, we prepared stable cell lines for TREK-1, TWIK-related acid-sensitive K^+^ channel (TASK-1), strongly inwardly rectifying K^+^ channel (Kir2.1), and human ether-a-go-go-related gene (HERG-1) channels and transiently expressed other channels in 293T cells using a calcium-phosphate transfection method. For the establishment of the stable lines, we used lentiviral vectors, and the preparation methods for the lentiviral vectors were described in our previous report [16]. TR-1 cells, which stably expressed TREK-1 channel and were grown on a small cover glass (3 × 18 mm), were placed in a recording chamber with an internal volume of 200 μl. Whole-cell currents were recorded in Tyrode solution using an Axopatch 200B amplifier (Axon Instruments, Foster City, CA) at 25°C. Tyrode solution contained (in mM): NaCl 140, KCl 5.4, NaH_2_PO_4_ 0.33, CaCl_2_ 2, MgCl_2_ 1, HEPES 5, and glucose 5.5 (pH 7.4 adjusted with NaOH). Patch pipettes pulled from borosilicate glass (Narishige, Tokyo, Japan) were filled with an internal solution containing (in mM): K-aspartate 66, KCl 71.5, KH_2_PO_4_ 1, EGTA 5, HEPES 5, and MgATP 3 (pH 7.4 adjusted with KOH). Recordings were digitized at 10 kHz, and low-pass filtered at 2 kHz. Step pulses were applied from a holding potential of -70 mV. Test compounds were initially dissolved in dimethyl sulfoxide (DMSO) at a higher concentration (100 mM) and then diluted with Tyrode solution by mixing vigorously for several minutes. After the run-up ended, Tyrode solution containing a test compound (200 μl) was added to the chamber using a pipette over 10 seconds. Perfusion (100 μl/min) was continued to maintain the water level throughout recordings. pH and osmotic pressure of solutions were measured with a pH meter (Horiba, Kyoto, Japan) and a freezing point osmometer (Knauer, Berlin, Germany), respectively.

### Behavioral tests

Male ICR mice (n = 317 in total) aged 5 weeks and weighing 32 ± 2 g were purchased from Shimizu Laboratory Supplies (Kyoto, Japan). For the social defeat stress test, we used 28 C57BL/6J mice (5 weeks old, Shimizu Laboratory Supplies) and 30 ICR mice (7–8 weeks old, Charles River Labs Japan, Yokohama, Kanagawa, Japan). Mice were maintained for at least 1 week prior to experiments. Animals were kept in groups in plastic cages. They had free access to food and water, and were kept in a 12-h light/dark cycle (08:00–20:00). Experiments were carried out between 10:00 and 18:00 in a quiet and air-conditioned experimental room, in which mice were habituated for at least 30 min before each experiment. Animal experiments were performed in accordance with the guidelines of the Physiological Society of Japan and were approved by the Committees on Animal Experiments at Kansai Medical University and the Kurashiki University of Science and the Arts. Ostruthin was initially dissolved in DMSO at higher concentration (100 mM) and then diluted in phosphate-buffered saline (PBS). The diluted solution was mixed vigorously for more than 10 min before the experiments, and an equal volume of DMSO was added to PBS as a control. PBS and ostruthin were administered intraperitoneally 30 min before mice behavioral tests (0.01 ml/g body weight). Each animal was used only once, except for the wheel running test, which was carried out immediately after elevated plus maze.

### Open-field test

The open-field test was performed in accordance with a previous report [17] with minor modifications. The open-field consisted of a square (90 × 90 cm), in which the floor was divided into 16 equal squares. After a mouse was placed in a corner of the open field facing the wall, the number of the entries into the central squares, and whole line crossings were counted for 5 min. After removal of animal, the apparatus was cleaned and wiped.

### Elevated plus maze

The elevated plus maze apparatus consisted of four arms set in a cross pattern from a neutral central square (6 × 6 cm). Two opposite arms were delimited by vertical walls (closed arms, 30 × 15 × 6 cm), whereas the other two arms had unprotected edges (open arms, 30 × 6 cm). The maze was elevated 40 cm above the floor. At the beginning of each 5 min test session, a mouse was placed in the central neutral area, facing one of the open arms. The total number of the entries to open arms and time spent in the open arms were measured. After removal of the animal, the apparatus was cleaned and wiped.

### Light/Dark box

The light/dark box consisted of two compartments with a total exterior size of 46 × 27 × 30 cm. One-third of the box was used as a dark compartment, which was covered with a black wall and lid, and the rest of the area was used as a light compartment. These compartments were connected via a small opening (7 × 7 cm), enabling transition between the two compartments. We placed a mouse in the center of the light area and measured the time spent in the light area and the number of transitions between the light and dark compartments for 5 minutes.

### Forced swim test

The forced swim test was performed in accordance with a method reported previously [18]. Briefly, mouse was placed into a 2 L glass beaker containing 12 cm of water (25–27°C). Mouse was allowed to swim for 6 min and the activity was measured. The duration of immobility is defined as the absence of activity, such as escape-oriented behaviors. The last 4 min of data were used for analysis. A mock experiment was performed 1 day before the real test.

### Tail suspension test

The tail suspension test was performed in accordance with a method reported previously [19]. The mouse was hung on a hook using adhesive tape placed 2 cm from the extremity of its tail 50 cm above the table. The amount of time spent immobile was recorded for 5 min.

### Social defeat stress test

Social defeat stress test was carried out according to a previous report [20]. A test mouse (C57BL/6J) was exposed to an aggressor mouse (ICR) for 10 min, and then these mice were maintained in the same cage but separated by a plastic plate with small holes. The next day, C57BL/6J mouse was exposed to another unfamiliar aggressor mouse, and this was repeated 9 times in total. On the 10th day, the test mouse was placed in an open-field arena, which has a wire-mesh enclosure for social interaction testing. The target zone (14 cm × 24 cm) encompasses a rectangular area around the wire-mesh enclosure. Time spent in the target zone was measured firstly with the empty mesh enclosure for 150 sec. After an interval of 30 sec, the second test was done with an unfamiliar ICR mouse present in the enclosure. The time spent in the target zone with empty enclosure was subtracted from that with the aggressor mouse.

### Wheel running test

Immediately after the elevated plus maze test, the same mouse was subjected to the voluntary wheel running test. The number of rotations was measured for 5 min using an activity monitoring running wheel (Muromachi Kikai, Tokyo, Japan).

### Anti-c-Fos immunostaining

The mouse was put into an anesthetizing jar and isoflurane (>0.2 ml) was added to the jar. After confirming that mouse was deeply anesthetized, its brain was fixed by transcardiac perfusion with 4% paraformaldehyde in PBS. The brains were sliced coronally (100 μm in thickness) using a microslicer (PRO7, Dosaka, Kyoto, Japan). The free-floating slices were reacted with an anti-c-Fos antibody (1: 200, goat, Santa Cruz, Dallas, TX) dissolved in PBS containing 0.3% BSA and 0.3% Triton-X-100, at 4°C for 48 h. The immunoreaction was visualized using a secondary antibody (rabbit anti-goat IgG, Vectastain Elite ABC-HRP Kit, Vector Labs, Burlingame, CA) and 3,3'-Diaminobenzidine (DAB, Tokyo Chemical Industry, Tokyo, Japan) at room temperature in accordance with the manufacturer’s instructions.

For statistical analysis, the number of c-Fos-immunoreactive cells was quantified using the Image-J software. Briefly, fields (0.16 mm^2^) of c-Fos immunoreactivity were acquired by a microscope IX70 (Olympus) using a CCD camera. DAB-positive nuclei, of which diameters were longer than 7 μm, were considered as anti-c-Fos-immunoreactive neurons. The immunoreactive cell numbers were normalized to cells/mm^2^.

### Statistical analysis

Data are given as the mean ± SEM. The normality of data distribution was confirmed with the Shapiro-Wilk test. Statistical significance between two groups was determined using Student’s t-test. Those obtained from three or more groups were analyzed statistically by one-way analysis of variance (ANOVA) followed by Bonferroni test. A *p* value of <0.05 was considered significant. The number of asterisks indicates the *p* values: *, *p*<0.05; **, *p* < 0.01; ***, *p*<0.005.

## Results

### Activation of TREK-1 channels by ostruthin

**Fig 1:**
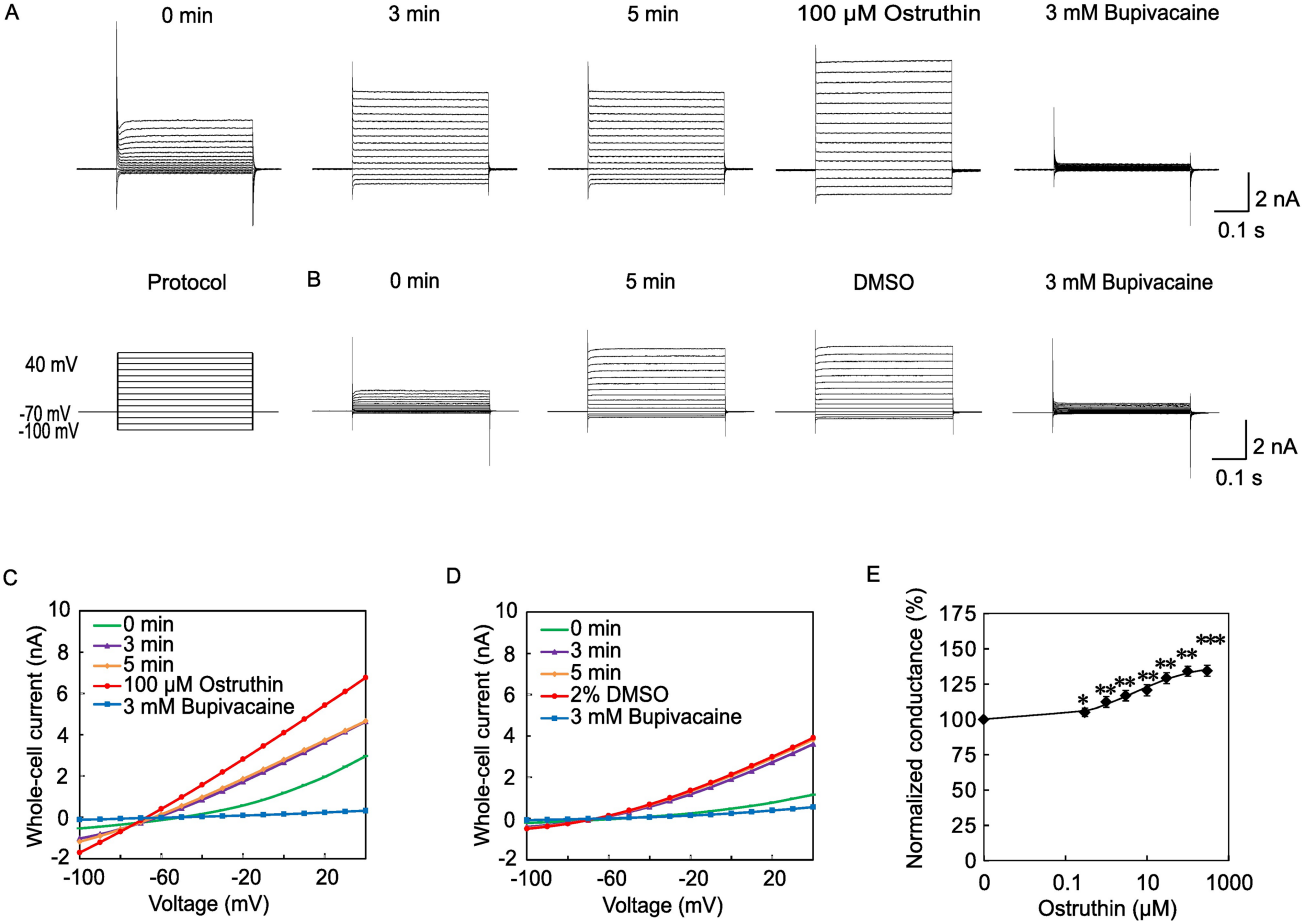
Activation of TREK-1 currents by ostruthin. (A) Run-up and ostruthin-induced increases in whole-cell TREK-1 currents. Step-pulses, which are indicated in the protocol, evoked outward rectified currents immediately after whole-cell access was made (0 min). Because of the run-up, the whole-cell TREK currents increased gradually and reached a plateau at 3 min, without further increase by 5 min. Application of 100 μM ostruthin increased TREK currents (100 μM ostruthin). Application of 3 mM bupivacaine blocked the currents nearly completely. (B) DMSO had no effect, serving as a negative control. (C and D) The current-voltage relationship of the whole-cell TREK-1 currents recorded from ostruthin- and DMSO-treated cells. The whole cell currents were increased from 0 to 3 min and stayed the same at 5 min. The current was increased by the addition of ostruthin and that was completely inhibited by bupivacaine. Contrastingly, DMSO had no effect on the current. (E) The concentration-response curve of ostruthin. The EC_50_ was found to be 5.3 μM. (n = 5).

We previously prepared a lentiviral vector that expressed the TREK-1 channel [16] and using this vector we established a cell line, TR-1, which stably expressed the channel [21]. First, we confirmed the expression of the channel current in this cell line with whole cell patch-clamp recordings. Immediately after whole-cell access, small outward currents were recorded in response to depolarizing step pulses (Fig 1A, 0 min). As reported previously [21], the channel current exhibited a gradual and spontaneous increase, which is called run-up, and reached a maximum level 3 min after establishing the whole-cell access. After the end of run-up (5 min), the current amplitude was the same as at 3 min (Fig 1A and 1C). Therefore, in the following screening assays, test compounds were applied 5 min after the establishment of whole-cell access.

We tested dozens of botanical compounds and found that a hydroxyl coumarin-derivative, ostruthin, had TREK-1 channel activating activity; the application of compounds increased the current even after the end of the run-up in a dose-dependent manner (Fig 1A, 1C and 1E). The increased current after the application of ostruthin was inhibited nearly completely by a TREK-1 selective blocker, bupivacaine (3 mM), suggesting that the increased current was attributable to the channel. The EC_50_ of activation was 5.3 μM.

**Fig 2:**
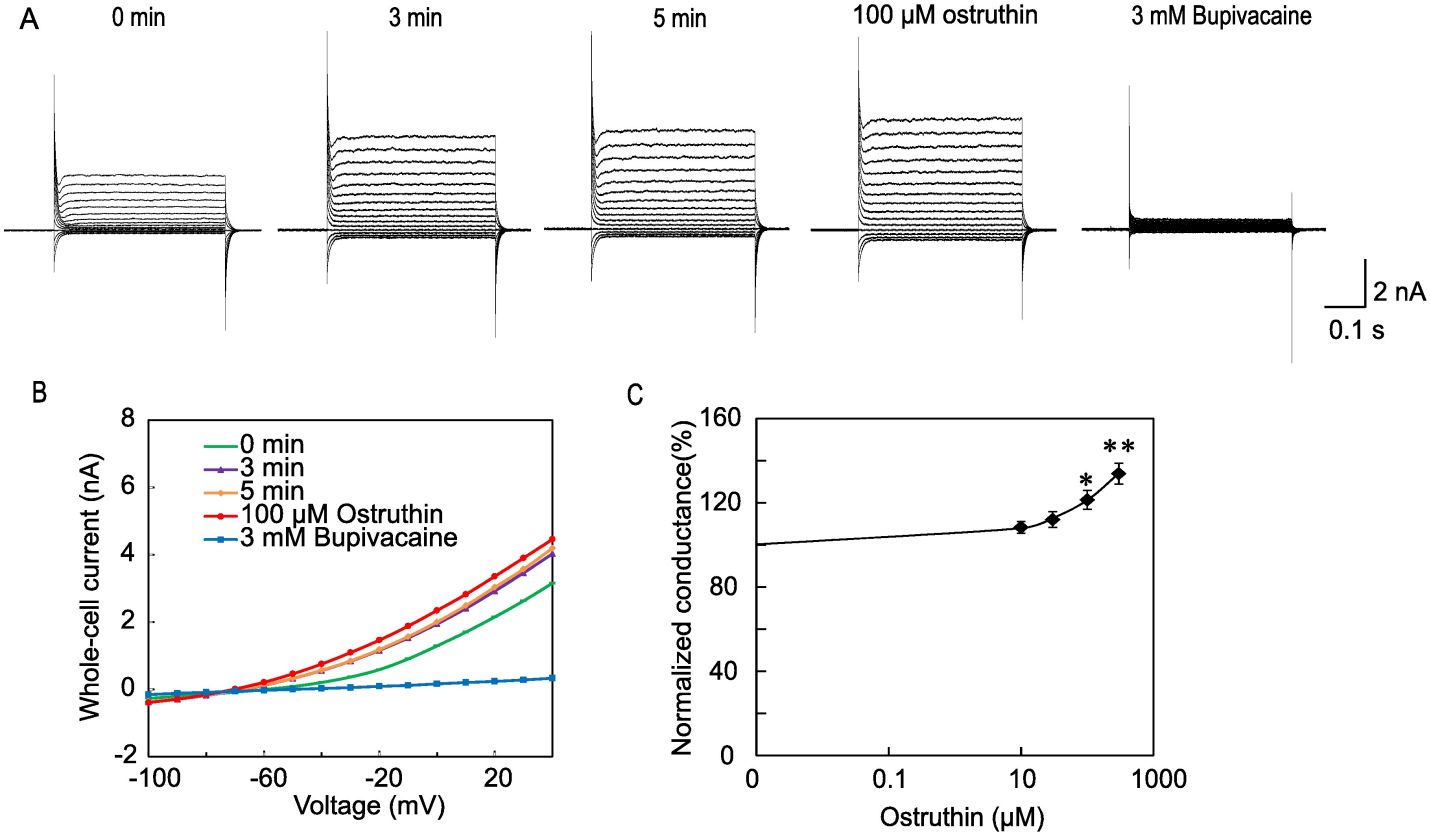
Activation of TREK-2 currents by ostruthin. (A and B) Run-up and activation of TREK-2 channel currents. Whole cell TREK-2 channel currents expressed in 293T cells showed a run-up, which ceased within 3 min. Ostruthin, which was applied 5 min after the whole-cell access, activated TREK-2 channel currents at 100 μM. The activated current was inhibited by bupivacaine nearly completely. (C) The concentration-response curve of ostruthin on the TREK-2 currents. The EC_50_ was found to be 3.7 mM. (n = 5).

TREK-1 channels are known to be activated by low pH and mechanical stretch [22]. Therefore, it is possible that the addition of ostruthin containing solution activated the channel through the changes in pH or osmotic pressure. To test these possibilities, we measured the pH of Tyrode solution before and after addition of 100 μM ostruthin, and observed no significant change (from 7.31 ± 0.17 to 7.33 ± 0.22, *p* = 0.645, paired *t*-test, n = 6). The addition of ostruthin, which was initially dissolved in DMSO at 100 mM, to a final concentration of 100 μM significantly increased the osmotic pressure (from 284.0 ± 11.8 to 297.0 ± 16.8 mOsm/L, n = 6, *p* < 0.01, paired *t*-test). This increase in the osmotic pressure was simply due to the DMSO. Indeed, addition of an equivalent amount of DMSO resulted in the similar increase (from 280.5 ± 10.5 to 297.2 ± 17.5 mOsm/L, n = 6, *p* < 0.01 paired *t*-test). To exclude the possibility that DMSO and accompanying increase in the osmotic pressure had the activating effect on TREK-1 channel, we applied DMSO up to 2%. We found no effect on the TREK-1 current (Fig 1B and 1D), excluding the possibility that the activation was caused by the mechanical stretch. Ostruthin increased the current at all voltages tested (Fig 1C), suggesting voltage-independency of the activation. We next tested the effect of ostruthin on TREK-2 channel, which was transiently expressed in 293T cells. Ostruthin activated the TREK-2 channel current at higher concentrations (EC_50_ 3.7 mM) compared with TREK-1 (Fig 2). TREK-2 channel was reported to be insensitive to bupivacaine at 100 μM [23], but addition of a high concentration of bupivacaine (3 mM) almost blocked the current. This result suggests that TREK-2 was slightly sensitive to bupivacaine and that the increased current was attributable to the TREK-2 channel current, not to the deterioration of the patch-clamp recording condition.

### Ostruthin inhibited other K^+^ channels

**Fig 3:**
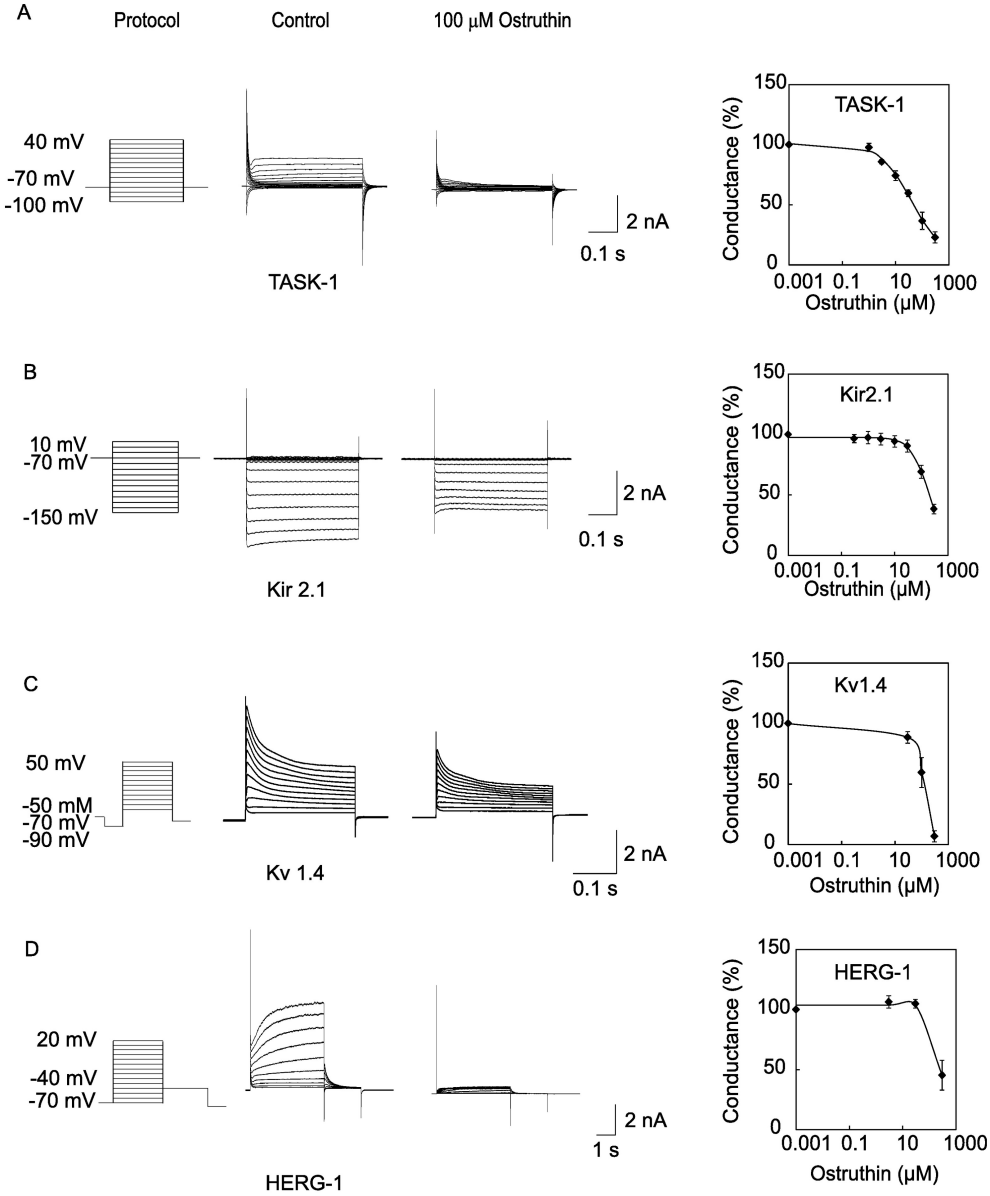
Inhibitory effect on other K^+^ channels. (A) Inhibition of TASK-1 currents. Whole cell TASK-1 currents, expressed in a 293T cell line, were evoked by step-pulses, as indicated in the protocol. The application of 100 μM ostruthin inhibited TASK-1 currents in a concentration-dependent manner (n = 10). (B) Inhibition of Kir2.1 currents. Kir2.1 currents were evoked the step pulses, and application of 100 μM ostruthin decreased the currents by approximately 60% (n = 5). (C) Inhibition of Kv1.4 currents. Kv1.4 channel currents expressed in a 293T cells were evoked by depolarizing step pulses with a preceding hyperpolarizing pulse. Ostruthin inhibited the currents (n = 5). (D) Inhibition of HERG-1 currents. HERG-1 currents were measured as the outward tail current after the depolarizing step pulses, as shown in the protocol (n = 5).

To examine the effect of ostruthin on other K^+^ channel families, we made whole-cell recordings from cells expressing TASK-1 and -3, Kir2.1, Kv1.4, HERG-1 and -3, KCNQ-1 and -3, and applied the compound to cells expressing these channels. Ostruthin inhibited these channels at higher concentrations in a concentration-dependent manner (Fig 3A–3D). The IC_50_ values of these channels were: 41 μM (TASK-1, Fig 3A), 227 μM (TASK-3), 198 μM (Kir 2.1, Fig 3B), 269 μM (Kv1.4, Fig 3C), and 216 μM (HERG-1, Fig 3D). Ostruthin had no effect on KCNQ-1 and -3 or HERG-3 channels at 100 μM (data not shown). These results suggested a weak and general inhibitory effect on K^+^ channels.

### Anxiolytic and antidepressive effects of ostruthin

**Fig 4:**
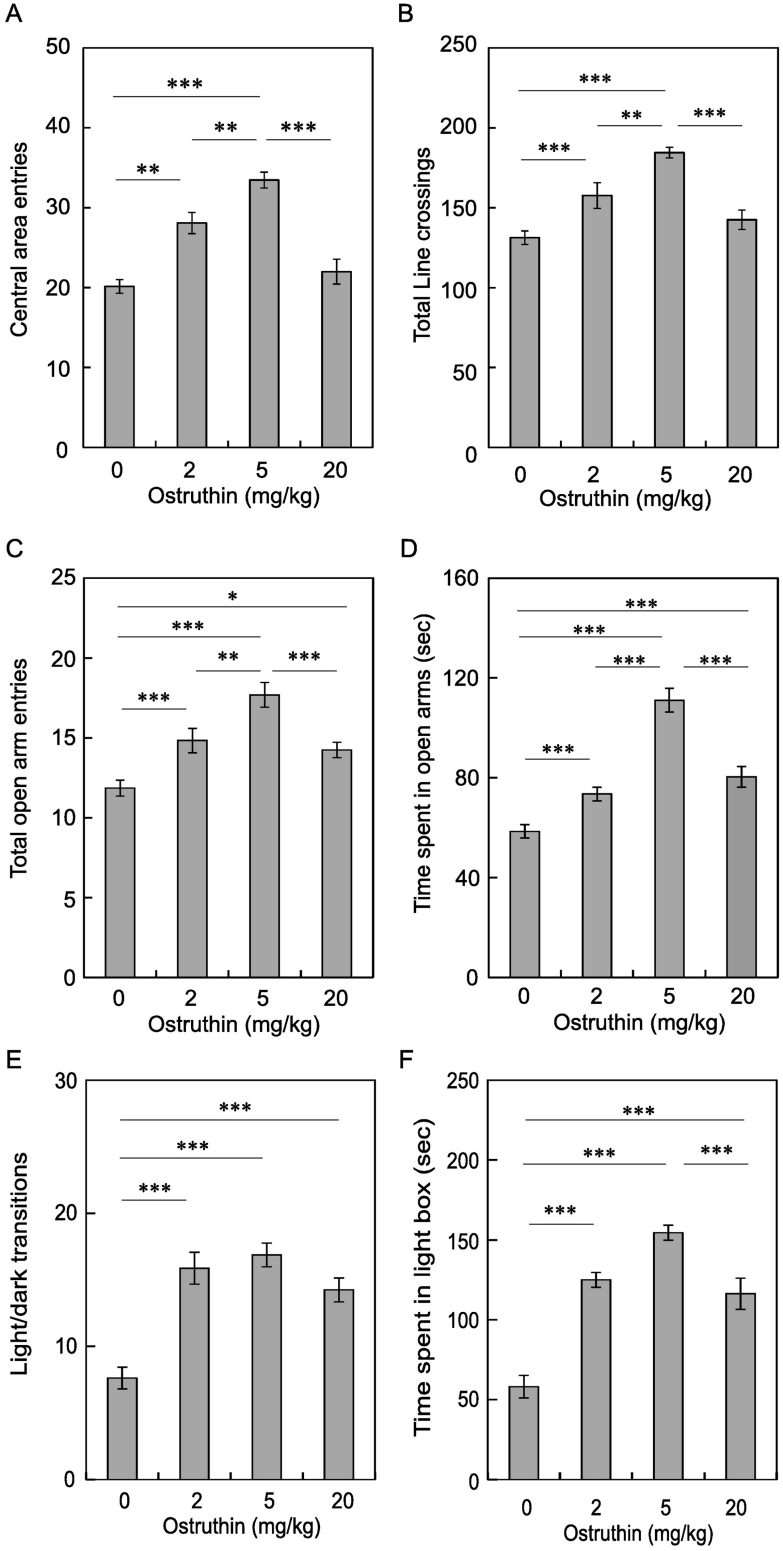
Anxiolytic effect of ostruthin. (A and B) Effect of ostruthin on the anxiety-like behavior of mice was examined using the open field test. Total numbers of entries into the central area (A) and line crossings (B) made in 5 minutes were recorded. (C and D) Anxiolytic effect in the elevated plus maze. The number of entries into the open arms (C) and time spent in the open arms (D) in 5 minutes were measured. (E and F) Anxiolytic effect in the light/dark box test. The number of transitions between the light and dark boxes (E) and time spent in the light box (F) in 5 minutes were measured. All the experiments were done 30 min after the administration of PBS (0) or the indicated dose of ostruthin.

Reportedly, mice lacking the TREK-2 gene showed a gender-dependent change in anxiety-like behavior [24]. In addition, riluzole is generally considered as a blocker for Na^+^ channels and glutamate receptors, but it can activate TREK-1 channels [9]. Riluzole was also shown to have anxiolytic and antidepressive effects in humans [8, 25]. Therefore, we tested the effect of ostruthin on mice anxiety-like behavior in open-field, elevated plus maze, and light/dark box tests. We administered 0, 2, 5 and 20 mg/kg of ostruthin 30 min before tests. In the open-field test, one-way ANOVA analysis revealed that the number of entries to the central area was significantly different depending on doses of ostruthin (F(3, 44) = 21.3, *p* < 0.00001, n = 12, Fig 4A). The entries were significantly increased by administration of lower doses of ostruthin (2 and 5 mg/kg, *p* < 0.01 and 0.0001, vs 0 mg/kg, post hoc Bonferroni test, n = 12). Total line crossings were also changed similarly depending on doses (F(3, 44) = 12.6, p < 0.00001, Fig 4B), and were increased by the lower doses (2 and 5 mg/kg, *p* < 0.0001, vs 0 mg/kg, n = 12). If ostruthin (5 mg/kg) was equally distributed throughout the mouse body, the concentration was estimated to be 17 μM, which was higher than the EC_50_ for TREK-1 but much lower than the IC_50_s for other K^+^ channels. Of note, a higher dose (20 mg/kg) of ostruthin decreased the anxiolytic effect, namely the central area entries were significantly decreased (*p* < 0.0001, vs 5 mg/kg), resulting in a bell-shaped dose-response relationship. A similar decrease in total line crossings was observed with the higher dose (*p* < 0.0001, vs 5 mg/kg).

This anxiolytic effect with the bell-shaped dose-response relationship was also observed in the elevated plus maze and light/dark box tests. In the elevated plus maze, significant difference was revealed by one-way ANOVA (F(3, 53) = 18.0, *p* < 0.00001, n = 12 and 21 (only for 0 mg/kg), Fig 4C). Lower doses of ostruthin (2 and 5 mg/kg) increased the number of entries to the open arm (*p* < 0.005 and 0.0001, Bonferroni test, vs 0 mg/kg). Higher dose (20 mg/kg) decreased the entry to the open arm (*p* < 0.005, vs 5 mg/kg), resulting in a bell-shaped dose-response relationship (Fig 4C). Similarly, the time spent on the open-arm was changed in the same way (F(3, 53) = 47.9, *p* < 0.00001, Fig 4D). The time was decreased with the highest dose (*p* < 0.0001, vs 5 mg/kg).

Consistent results were also observed in the light/dark box test (F(3, 28) = 18.6, *p* < 0.00001, ANOVA, n = 8, Fig 4E), in which ostruthin increased the number of transitions between the light and dark boxes (*p* < 0.001, post hoc Bonferroni test). The transition number was non-significantly decreased with the highest dose. The time spent in the light box was changed by ostruthin similarly (F(3, 28) = 34.6, *p* < 0.00001, n = 8). Osturuthin treatment significantly increased the time (*p* < 0.0001, vs 0 mg/kg). Again the time spent in the light box was decreased with the administration of 20 mg/kg of ostruthin (*p* < 0.0005, vs 5 mg/kg), resulting in a bell-shaped dose–response relationship (Fig 4F). These data consistently showed the anxiolytic effect of ostruthin.

**Fig 5:**
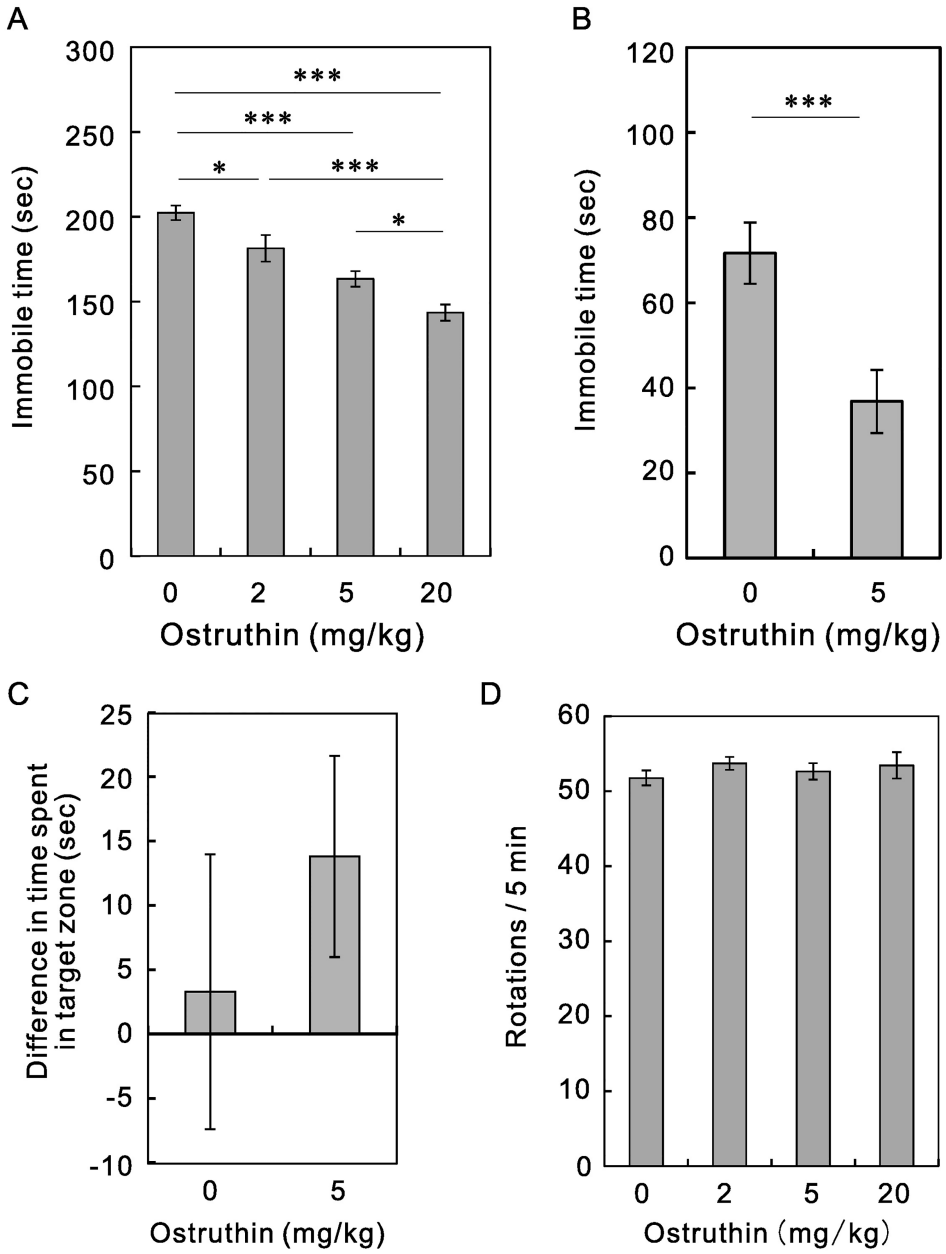
Antidepressive effect of ostruthin. (A) Effect of ostruthin on the depression-like behavior of mice was examined using the forced-swim test. The amount of time spent immobile in the last 4 min of the swimming period (6 min) was recorded. Administration of ostruthin 30 min before test significantly reduced the time spent immobile in a dose-dependent manner. (B) Antidepressive effect of ostruthin in the tail-suspension test. The amount of time spent immobile was recorded for 5 min. (C) Insignificant effect on social defeat stress test. After the exposure to aggressor ICR mice for 9 days, C57BL/6J test mouse was placed in an open-field arena. Firstly the time spent around the empty enclosure and subsequently that with an unfamiliar ICR mouse was measured. The former duration was subtracted from the later. (D) Lack of effect on voluntary motility. After the end of the elevated plus maze, mice were subjected to voluntary wheel running test for 5 min. All experiments were carried out 30 min after the administration of PBS (0) or the indicated dose of ostruthin.

Reportedly, TREK-1-deficient mice showed a depression-resistant phenotype through the activation of neurons in the DRN [6]. Because ostruthin activates TREK-1 channels, it is possible that ostruthin has the opposite effect on depression-like behavior. To test this, ostruthin-treated mice were subjected to forced swim and tail suspension tests. Unexpectedly, ostruthin showed an antidepressive effect in both tests. In the forced swim test, the time spent immobile was decreased by ostruthin (F(3,28) = 20.5, *p* < 0.00001, n = 8, Fig 5A). The decrease in immobile time was dose-dependent. Similarly, administration of ostruthin decreased the time spent immobile in the tail suspension test (*p* < 0.005, Student’s t-test, n = 14, Fig 5B). We also tested the antidepressive effect of social defeat stress, and osturuthin increased the time difference in the interaction area non-significantly (*p* = 0.440, Student’s *t*-test, n = 14, Fig 5C).

These data consistently suggest the antidepressive effect of ostruthin with the decrease in the immobile time. But it is also possible that these effects are attributable to an increase in general locomotor activity. To exclude this possibility, mice used for the elevated plus maze were subjected to voluntary wheel-running test immediately after the elevated plus maze test (Fig 5D). Ostruthin had no effect at any dose (F(3,24) = 0.514, *p* = 0.68, n = 7), suggesting the unlikelihood of this possibility.

**Fig 6:**
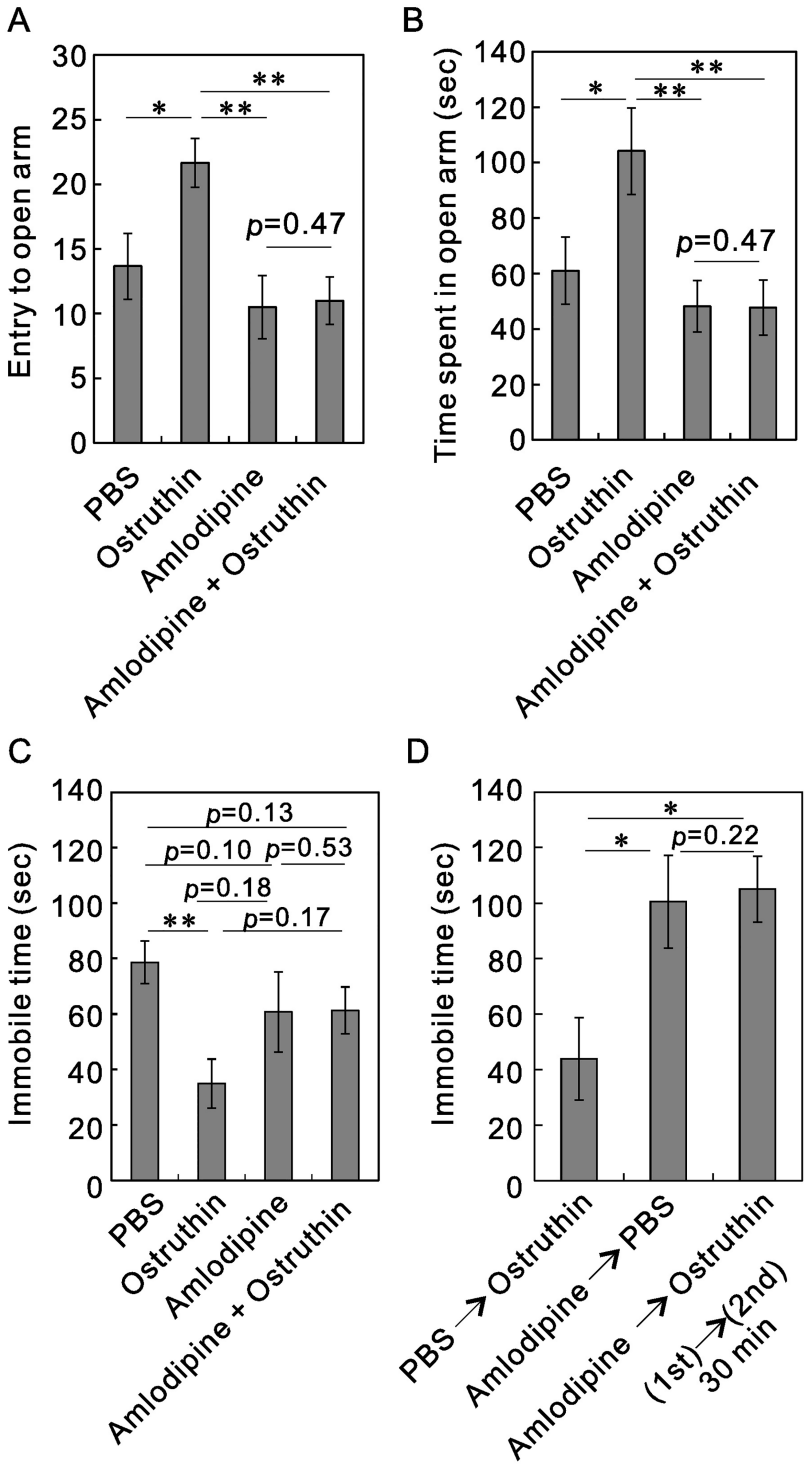
Effect of co-administration of amlodipine on the anxiolytic and antidepressive effects of ostruthin. (A and B) Blockade of the anxiolytic effect of ostruthin by co-administration of amlodipine in the elevated plus maze. Mice were administrated the compounds 30 min before the test. Although administration of ostruthin increased the number of entries into the open arm (A) and the time spent on the open arm (B), co-administration with amlodipine blocked the increase. (C) Blockade of the antidepressive effect of ostruthin by co-administration of amlodipine in the tail suspension test. Although administration of ostruthin decreased the immobile time, it did not do so in the presence of amlodipine. (D) Pre-administration of amlodipine effectively blocked the antidepressive effect of ostruthin compared with pre-administration of PBS.

To confirm that TREK-1 channels are involved in these anxiolytic and antidepressive effects, we administrated ostruthin (5 mg/kg) together with amlodipine (10 mg/kg). Amlodipine is generally used as a Ca^2+^ channel blocker, but it can block TREK-1 channels too [26]. The effect of co-administration was examined using the elevated plus maze and the tail suspension tests. If ostruthin exhibited anxiolytic and antidepressive effects through the activation of TREK-1 channels, the co-administration of amlodipine should block these effects. If amlodipine was equally distributed in the whole body, the dose of 10 mg/kg would be equivalent to 18 μM, which is higher than the reported IC_50_ for TREK-1 channel currents (0.43 μM) [26]. One-way ANOVA analysis revealed that the number of entries to the open arm was significantly different between four groups (F(3,20) = 5.64, *p* < 0.01, n = 6, Fig 6A). Whereas administration of ostruthin increased the number of entries to the open arm (*p* < 0.05, vs PBS, post hoc Bonferroni test, n = 6), the administration of amlodipine non-significantly decreased them (*p* = 0.10, vs PBS). We then co-administrated ostruthin and amlodipine to mice. As expected, in the presence of amlodipine, ostruthin did not increase the number of entries to the open arm (*p* = 0.47, vs amlodipine, Fig 6A). The number of entries to the open arm was significantly decreased by co-administration of amlodipine (*p* < 0.01, vs ostruthin). Similarly, One-way ANOVA analysis revealed that the time spent in the open arm was significantly different between four groups (F(3,20) = 5.31, *p* < 0.01, n = 6, Fig 6B). In the absence of amlodipine, ostruthin increased the time spent in the open arm (*p* < 0.05, PBS vs ostruthin, n = 6, Fig 6B), whereas it did not in the presence of amlodipine (*p* = 0.47). The co-administration of amlodipine significantly decreased the time spent in the open arm (*p* < 0.01, vs ostruthin). These results suggest the involvement of TREK-1 channels in the anxiolytic effect of ostruthin.

This is the case even for the antidepressive effect examined with the tail suspension test (Fig 6C). One-way ANOVA analysis revealed that the immobile time was significantly different between four groups (F(3,36) = 2.94, *p* < 0.05, n = 9 and 13 (only for ostruthin)). In the absence of amlodipine, the time spent immobile was significantly decreased by ostruthin-treatment (*p* <0.01, post hoc Bonferroni test, PBS vs ostruthin, n = 9 and 13). That of amlodipine-treated mice was non-significantly decreased (*p* = 0.10, vs PBS, n = 9). In the presence of amlodipine, ostruthin had no effect on the time spent immobile (*p* = 0.53), suggesting the involvement of TREK-1 channels in the antidepressive effect too. The co-administration of amlodipine with ostruthin increased the immobile time compared with ostruthin, but the difference was not significant (*p* = 0.17). This might be attributable to an insufficient blockade with co-administration of amlodipine. To test this, we administrated PBS or amlodipine 30 min before ostruthin administration and examined the antidepressive effect 30 min after the ostruthin injection (Fig 6D). One-way ANOVA analysis revealed that the immobile time was significantly different between three groups (F(2,21) = 5.42, *p* < 0.05, n = 8). As expected, preceding administration of amlodipine significantly increased the immobile time compared with preceding PBS administration (*p* < 0.05, PBS→ostruthin vs amlodipine→ostruthin, n = 8), suggesting the efficient blockade with preceding administration of amlodipine. After the pre-administration of amlodipine, the secondary ostruthin administration was ineffective, namely the immobile time was similar to that of the secondary PBS-administration (*p* = 0.22).

### Effect of ostruthin on anti-c-Fos immunoreactivity of stressed-mouse brains

The above findings raised a question about which nucleus is involved in these behavioral effects, on the assumption that ostruthin changed the neuronal activity of a nucleus and thereby changed the behavior of the mice. Is it the same as current drugs? To determine the nucleus involved, through which ostruthin exhibited its anxiolytic and antidepressive effects, we examined the effect of ostruthin on anti-c-Fos immunoreactivity, which is a marker of neuronal activity [27], in anxiety and depression related nuclei. We first examined the immunoreactivity in non-stressed animals, which only received PBS or ostruthin administration and were fixed 30 min after. Modest anti-c-Fos immunoreactivities were observed in some nuclei, but there was no difference between PBS- and ostruthin-treated mice (data not shown), suggesting that ostruthin had little effect on basal neuronal activity.

**Fig 7:**
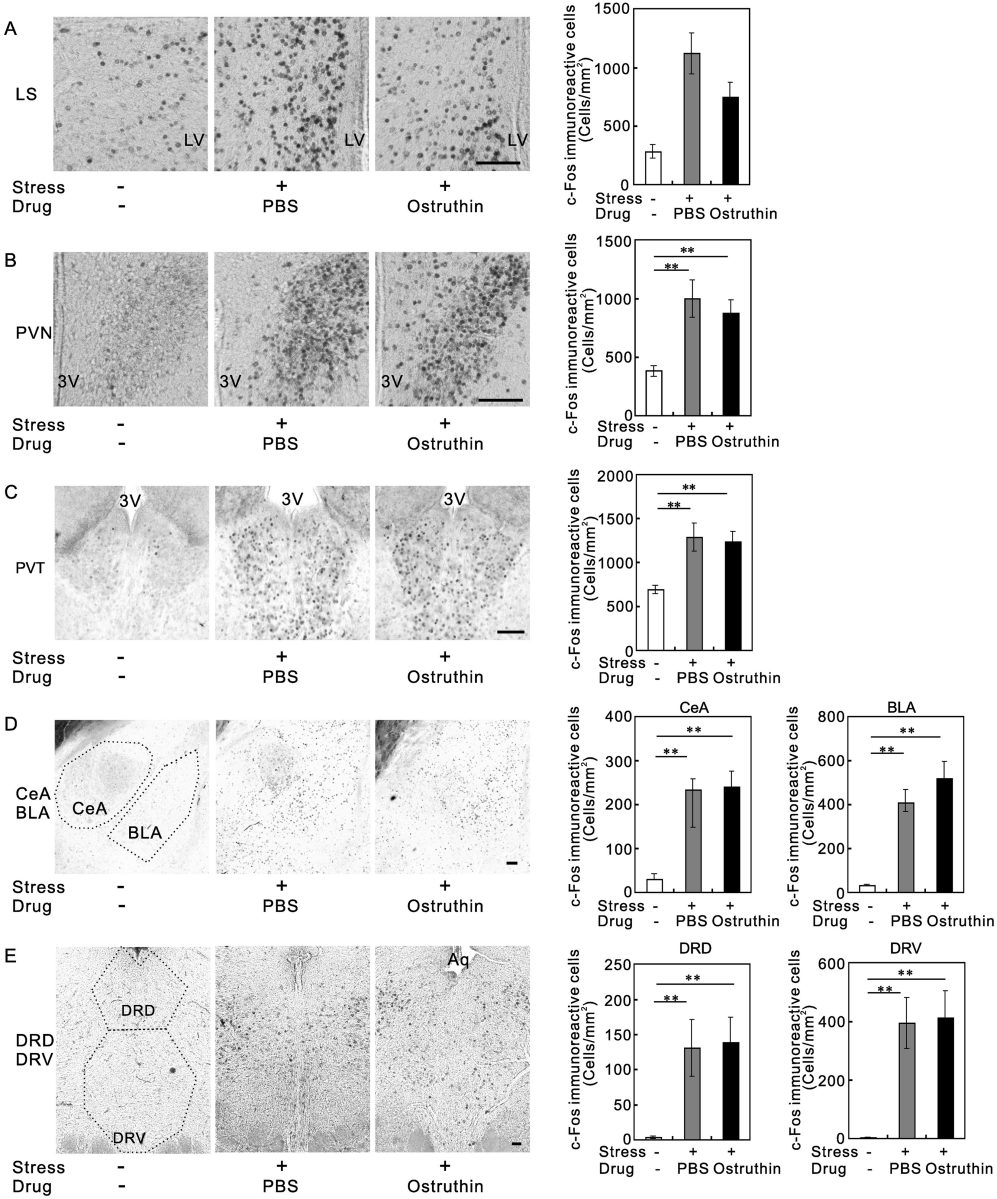
Stress-induced increase in anti-c-Fos immunoreactivity and suppression by ostruthin in the LS. (A) Anti-c-Fos immunoreactivity in the LS of non-treated and stressed mice. Anti-c-Fos-immunoreactivity was increased by stress-treatment, and the increase was suppressed by ostruthin. (B-E) The numbers of anti-c-Fos-immunoreactive cells were also increased by stress-treatment in the hypothalamic paraventricular nucleus (B), thalamic paraventricular nucleus (C), central and basolateral amygdala (D), and dorsal and ventral dorsal raphe nuclei (E). Ostruthin did not suppress the increase in these nuclei. LV indicates the position of the lateral ventricle; 3V, third ventricle; Aq, central aqueduct; CeA, central amygdala; BLA, basolateral amygdala; DRD, dorsal raphe nuclei; DRV, ventral raphe nuclei. (ANOVA followed by Bonferroni test; *, *p*<0.05; **, *p* < 0.01; ***, *p*<0.005; n = 4; Bar, 100 μm).

We then examined anti-c-Fos immunoreactivity 30 min after mild stress, i.e., being subjected to the tail suspension test. The mild-stress of the tail suspension test significantly increased anti-c-Fos immunoreactivity in stress-related nuclei, i.e. the lateral septum (LS, F(2,9) = 10.6, *p* < 0.005, n = 4, Fig 7A), hypothalamic paraventricular nucleus (PVN, F(2,9) = 12.7, *p* < 0.005, Fig 7B), thalamic paraventricular nucleus (PVT, F(2,9) = 4.38, *p* < 0.05, Fig 7C), central amygdala (CeA, F(2,9) = 19.9, *p* < 0.001) and basolateral amygdala (BLA, F(2,9) = 19.5, *p* < 0.001, Fig 7D), and dorsal (DRD, F(2,9) = 5.88, *p* < 0.05) and ventral nuclei of the dorsal raphe (DRV, F(2,9) = 10.1, *p* < 0.005, Fig 7E). We counted the number of immunoreactive neurons in these nuclei and found that these were significantly increased in response to the stress. We then examined the effect of ostruthin (5 mg/kg) on the stressed mice and found that there were no significant differences in most nuclei (Fig 7B–7E). However, only in the LS, ostruthin significantly suppressed the stress-induced increase in c-Fos immunoreactivity (Fig 7A).

## Discussion

### Effect of ostruthin on K^+^ channels

Previous reports showed that ostruthin has several biological activities, such as mitochondrial uncoupling [28], antimycobacterial activity [29], inhibition of smooth muscle cell proliferation [30], and inhibition of cell differentiation [31]. Here, we demonstrated that a botanical compound, ostruthin, activated TREK-1 channels at a low concentration, and TREK-2 channels at a higher concentration. Because K^+^ ions are essential for plant cell growth, osmoregulation, and stomatal opening, K^+^ ions absorbed from the soil are transported to the stems and leaves. For this reason, plant cells express many kinds of K^+^ channels, including TREK-like channels, which are expressed in the plasma membrane and tonoplast [12]. Therefore, compounds that activate TREK channels might be useful for plants as natural enhancers of K^+^ ion transport. Our present results indicated that botanical compounds are a promising resource for the identification of new lead compounds that can act as K^+^ channel modifiers.

TREK-1 channels were activated by arachidonic acid, docosahexaenoic acid, riluzole, and inhalational anesthesia [22, 32]. Inhalational anesthesia, i.e., halothane, isoflurane, diethyl ether, and Chloroform, was shown to bind to the intracellular C-terminal domain [33]. Ostruthin has features of the cell membrane- and blood-brain barrier-permeability: 1) low molecular weight (298), 2) small number of hydrogen bond donor (2) and acceptors (2), 3) modestly high partition coefficient (5.6), 4) small polar surface area (50.4 Å^2^). These features closely matched Lipinski’s rule of five. Therefore, ostruthin might bind to the intracellular C-terminal domain and activate the channel. On the other hand, ostruthin inhibited most other K^+^ channels at higher concentrations. Ostruthin might bind to a site, of which amino acid sequences are similar among these ostruthin-sensitive K^+^ channels, i.e. K^+^ ion filter and pore domain.

### Anxiolytic and antidepressive effects of ostruthin

Ostruthin consistently showed an anxiolytic effect in open-field, elevated plus maze, and light/dark box tests at a low dose (5 mg/kg). The involvement of TREK-1 channels in this anxiolytic activity was suggested by the inhibition of the effect of ostruthin by the co-administration of amlodipine, a blocker of TREK-1 channels. It was reported that riluzole, an activator for TREK-1 channels [9], showed anxiolytic effects [34], although riluzole has various pharmacological activities, e.g. blockade of NMDA receptors and voltage-dependent Na^+^ channels and inhibition of G-protein-dependent signal transduction [8]. Activation of TREK-1 channels might be the common mechanism for the anxiolytic activity of riluzole and ostruthin at least in part. Of note, an increased dosage of ostruthin (20 mg/kg) reduced the effect and resulted in a bell-shaped dose-response relationship. This might be attributable to ostruthin activating TREK channels at low concentration but inhibiting other K^+^ channels at higher concentrations. Namely, low-dose ostruthin suppressed anxiety-related neurons by activation of TREK-1 channels, whereas high-dose ostruthin activated neuronal activity by the inhibition of other K^+^ channels.

Ostruthin also showed an antidepressive effect in forced swim and tail suspension tests. The involvement of TREK-1 channels was again confirmed by blockade of the behavioral effect by co-administration of amlodipine. A previous study showed the antidepressant properties of riluzole in patients with treatment-resistant depression [25], although the authors paid attention to the anti-glutamatergic effect of riluzole rather than the effect on TREK-1 channels. In addition, openers for another K^+^ channel, KCNQ, were shown to have an antidepressive effect [35]. Thus, the activation of K^+^ channels might be the mechanism for the antidepressive effects of these compounds.

The immobile times were decreased in the forced swim and tail-suspension tests, and motility was increased in the open-field test. Therefore, it is possible that these antidepressant-like behaviors may be attributable to the increase in general motility. Although we could not exclude this possibility, other data suggest the low likelihood of this possibility: 1) Lack of effect of ostruthin on wheel running, which reflect voluntary general motility. 2) An insignificant increase in the time spent in the target zone in social defeat stress test.

### Possible differences in the mechanism of action from current drugs

Current antidepressants inhibit the reuptake of serotonin and norepinephrine and thereby enhance their transmission. Previous studies reported the involvement of TREK-1 channels in depression-like behavior in mice through the activation of neurons in the DRN [6, 10, 36]; it was explained that inhibition of TREK-1 channels led to the activation of serotonergic neurons in the nucleus and the enhancement of serotonin release. Indeed, Heuteaux et al. observed increases in spontaneous firing rates in TREK-1-deficient mice [6]. A serotonin-specific reuptake inhibitor, fluoxetine, inhibited TREK-1 channels in the μM range; therefore, its behavioral effect was considered to be attributable to the inhibition of the channels in the DRN neurons in part [37]. In contrast, we found that an activator of TREK-1, ostruthin, showed an antidepressive effect. In addition, our anti-c-Fos immunostaining examination failed to detect ostruthin-dependent changes in neuronal activities of the DRN. Instead, ostruthin significantly suppressed the stress-induced increase in c-Fos immunoreactive cells only in the LS. Ostruthin might exhibit its antidepressive effect through this nucleus rather than the DRN.

The possible mechanism for the anxiolytic activity of ostruthin also seemed to be different from current anxiolytic drugs. A commonly used anxiolytic group of medicines, benzodiazepines, enhance inhibitory GABAergic transmission and thereby inhibit anxiety-related neurons in the limbic system including the amygdala [38]. However, ostruthin had no effect on the stress-induced increase in anti-c-Fos immunoreactivity in the amygdala, on which current anxiolytic agents act. Indeed, our preliminary data showed that ostruthin had no effect on the amplitude or frequency of GABAergic mIPSCS in the amygdala neurons at 100 μM. Instead, ostruthin suppressed stress-induced activation of the LS. Thus, ostruthin may exhibit anxiolytic effects via a different neural pathway from benzodiazepines.

The LS is known to be involved in the anxiety- and depression-like behaviors [39]. Furthermore, the LS was reported to be involved in freezing behavior and relay the information of stress to other nuclei [27]. The suggested role of the LS neurons in the anxiety-like behavior was controversial: some reports suggested that the LS suppresses anxiety behavior [39, 40], whereas others suggested that the nucleus promotes anxious behaviors [41]. In either case, it is commonly assumed that the LS relays the information of stress to other nuclei. Thus, the suppression of the LS by ostruthin would be an indication of suppression of the relay. Mental stress can lead to both anxiety and depression, and therefore it is expected that ostruthin is effective in humans, especially for the drug-resistant cases.

It remains unclear how ostruthin suppressed the increase in anti-c-Fos immunoreactivity in the LS without affecting the other nuclei. It is likely that ostruthin directly activated TREK-1 channels expressed in the LS and thereby suppressed the stress-induced increase in neuronal activity. Indeed, a modest expression of TREK-1 mRNA was shown in the LS [5]. Another possibility is that ostruthin might indirectly affect neuronal activity in another nucleus and thereby inhibit activity in the LS. Finally, ostruthin might exert these inhibitory effects through the blockade of other K^+^ channels. The last possibility is, however, unlikely because the IC_50_s for other K^+^ channels were in the hundreds of μM range, which were higher than the estimated concentration caused by the dosage of 5 mg/kg. Furthermore, the involvement of TREK-1 channels was indicated by the blockade of the anxiolytic and antidepressive effects by the co-administration of amlodipine. Further work is needed to clarify this mechanism.
